# Characterising ecosystem service provision by two morphologically distinct kelp species (*Laminaria hyperborea* and *Saccharina latissima*) and the biophysical drivers shaping these services: a systematic map protocol

**DOI:** 10.1186/s13750-026-00385-w

**Published:** 2026-04-21

**Authors:** Benjamin Hall, Patrick Eskuche-Keith, Yaofa Ren, Pippa Jane Moore, Clare Fitzsimmons, Fabrice Stephenson

**Affiliations:** School of Natural and Environmental Sciences, Newcastle University, Newcastle upon Tyne, UK

**Keywords:** Marine spatial management, Systematic map, Kelp beds, Marine conservation, Ecosystem services

## Abstract

**Background:**

Important ecosystem services (ES), defined by the Millennium Ecosystem Assessment as benefits people obtain from ecosystems, are provided by kelp forests (e.g. nutrient cycling, nursery sites for fisheries and habitat provision). However, kelp forest degradation is extensive and ongoing with 40–60% of kelp forests degraded over the past 50 years threatening ES provision. Management efforts often overlook species-specific biophysical drivers of service provision and the role of morphological diversity (e.g. stipitate or prostrate growth forms) in mediating ES. Effective marine spatial planning that incorporates ES provision requires understanding of these drivers, but current efforts are limited by poor spatial data on kelp distributions and ES provision. This systematic map aims (i) to catalogue and classify the ecosystem services that have been reported for *Laminaria hyperborea* and *Saccharina latissima* (two kelp species with contrasting growth forms), and (ii) to map what evidence exists on biophysical drivers associated with variation in these services.

**Methods:**

Following Collaboration for Environmental Evidence (CEE) guidelines, this systematic map will conduct a search of peer-reviewed literature from Web of Science, Scopus, and Google Scholar using terms for kelp species and ES, refined via the PO framework (Population, Outcome). Search string replicability will be validated using 10 benchmark articles. The screening process will utilize a three-stage process (remove duplicates, title and abstract screening and full text screening) which will prioritise studies quantifying ES provision (e.g. wave attenuation, biodiversity metrics, kelp farming) and/or biophysical drivers of ES provision for *Laminaria hyperborea* and *Saccharina latissima*. Data will be synthesized narratively and, where feasible, presented through tables, figures and geographic mapping that compares ES across growth form and biophysical drivers (e.g., light availability for primary productivity, turbidity for carbon sequestration). This systematic map aims to identify gaps in knowledge of kelp service provision, with the results highlighting kelp ES knowledge to guide future and current marine spatial planning.

**Supplementary Information:**

The online version contains supplementary material available at 10.1186/s13750-026-00385-w.

## Background

Kelp species occupy around a third of the world’s coastlines across 109 socio-political regions, forming both highly productive and biodiverse habitats [67, 84, 125]. These ecosystems support a wide range of ecosystem services (ES), defined by the Millennium Ecosystem Assessment (MEA) as the benefits humans derive from ecosystems [143]. For example, kelp forests contribute to carbon sequestration through the transport of organic carbon from surface waters into the deep ocean ([48]; Table 1). Other documented services include primary production [46, 96, 97, 101], storm protection [42, 90], nutrient bioremediation [119, 120, 123, 134], and habitat and fishery provision [28, 93, 124, 125, 126]. Although service provision varies by species and location, estimates of kelp forest valuation, at the global scale, is between US$465 and US$562 billion/year [43]. Given their ecological and economic significance, kelp forest management has become a priority for current and future research [50, 63, 75].

**Table 1:** Ecosystem services documented for kelp species, including service descriptions and key references

Define service group	Individual service	Definition	References identified
Provisioning	Direct kelp harvest	Kelp is harvested directly for human consumption, including food products (e.g. seaweed snacks, sushi wraps) and industrial uses (e.g. alginates, biofuels).	[56, 96, 98, 112, 137]
	Commercial Fisheries	Kelp forests provide habitat and nursery grounds for commercially important fish and shellfish species, supporting fisheries and aquaculture industries.	[11, 114, 126, 127, 130]
	Kelp derived materials	Kelp is processed into materials such as alginates, agar, and carrageenan, which are used in food, pharmaceuticals, cosmetics, and other industries.	[5, 115, 133]
	Materials from associated organisms	Kelp forests support the growth of other organisms (e.g., epiphytes, invertebrates) that are harvested for materials or products.	[88]
	Energy	Kelp biomass can be converted into biofuels (e.g., biogas, bioethanol) as a renewable energy source.	[112]
	Medicinal resources	Kelp biomass has been used for centuries in traditional medicine practises and is now valued in modern pharmacy (e.g. wound dressings, antiinflammation, Omega three and fatty acids vitamins).	[13, 115]
	Genetic resources	Kelp species possess unique genetic material that can be used in biotechnology, aquaculture, and conservation efforts.	[11, 126]
Regulating	Regulating Carbon emissions	Kelp forests sequester carbon dioxide through photosynthesis, storing carbon in biomass and detritus, which can be exported to deep-sea sediments, contributing to climate change mitigation.	[2, 48, 73, 99]
	Regulating Acidification	Kelp absorbs CO₂, reducing ocean acidification in local areas and improving conditions for calcifying organisms (e.g. shellfish, corals).	[42, 55]
	Coastal protection	Kelp forests attenuate wave energy, reducing coastal erosion and protecting shorelines from storm damage.	[86, 90, 104, 140]
	Sediment trapping on reef	Kelp forests trap and stabilize sediments, improving water clarity and reducing sedimentation on adjacent reefs.	[80]
	Costal erosion	By reducing wave energy and stabilizing sediments, kelp forests help prevent coastal erosion and maintain shoreline integrity.	[86]
	Nutrient filtration	Kelp absorbs excess nutrients (e.g. nitrogen, phosphorus) from the water, reducing eutrophication and improving water quality.	[24, 57]
	Pollution filtration	Kelp can absorb and sequester pollutants (e.g. heavy metals, microplastics), improving water quality and reducing contamination in marine ecosystems.	[6, 26]
	Increased dissolved oxygen	Through photosynthesis, kelp forests increase oxygen levels in the water, supporting aerobic marine life.	[72, 122]
	Increased water clarity	Kelp forests reduce turbidity by trapping sediments and absorbing particles, improving light penetration and supporting photosynthetic organisms.	[15, 64, 78]
Habitat	IUCN listed species	Kelp forests provide critical habitat for species listed as threatened or endangered by the IUCN, supporting their survival and recovery.	[7, 92]
	Charismatic species	Kelp forests support iconic and culturally significant species (e.g. sea otters, seals, sharks) enhancing biodiversity and ecotourism.	[16, 70]
	Species Richness	Kelp forests host a high diversity of marine species, including fish, invertebrates, and algae, contributing to overall marine biodiversity.	[77, 109, 127, 141]
	Endemism	Kelp forests support species that are unique to specific regions, contributing to local biodiversity and ecological uniqueness.	[52]
Cultural	Local recreation, Tourism, Aesthetic	Kelp forests attract divers, snorkelers, and eco-tourists, offering recreational opportunities and aesthetic value. They also inspire art, photography, and cultural appreciation of marine environments.	[69, 132]
	Sense of connection and place, myths and legends, traditional/indigenous use	Kelp forests hold cultural and spiritual significance for coastal communities, featuring in local traditions, myths, and indigenous practices.	[36, 137]
	Scientific knowledge generation	Kelp forests serve as natural laboratories for research and education, advancing scientific understanding of marine ecosystems and informing conservation efforts.	[121]

The inclusion of ES in management is well established as a tool for valuing ecosystems, with the MEA’s four ES categories (supporting, provisioning, regulating and cultural) aiming to promote equitable evaluation of services [89, 143]. Integrating ES into environmental management can help balance ecological health against human needs [94], particularly because ES provide a “common language” to facilitate stakeholder engagement and explore synergies and trade-offs [107]. The use of ES can encourage a holistic management approach, prioritizing long-term resilience over short-term gains [129], highlight underappreciated services [37, 58, 85], and discourage focus on individual service provision [10, 53, 108]. In addition, the use of ES for informing management of kelp dominated ecosystems is particularly relevant due to their wide spatial coverage [67, 117] and the extensive services they provide at spatial and temporal scales [9, 16, 46]. Despite the importance of kelp forests for providing ES, public and political awareness of kelp ecosystems remains low [79], likely in part due to the limited research funding compared to other marine ecosystems [79, 135, 136].

Kelp forest distribution is strongly influenced by a range of environmental factors including temperature, light, wave exposure, nutrients, suitable habitat, and grazing [19, 67, 102, 103]. Climate change is likely to directly alter many of these factors, and in turn, disrupt associated ecosystem services either temporarily or permanently [34, 47, 49, 87]. In extreme scenarios, climate change causes shifts to alternative ecosystem states that provide substantially different services [49, 139]. Additional threats to kelp forests include ocean warming [111], marine heatwaves [8], increased grazing pressure [138], and coastal industrialization. Subsequently, 40–60% of kelp forests have degraded over the past 50 years [121], including localised extinctions [49]. Kelp forests have declined in one-third of the world’s ecoregions [75], twice as fast as coral reefs and four times faster than tropical forests [45], which, in turn, is likely to have significantly reduced the quality and quantity of ES provided [50].

Understanding the potential impacts of marine environmental change on kelp ES in order to prioritise management strategies to mitigate these impacts, requires an understanding of the spatial distribution of ES, and how the nature of these services may be altered [50, 63, 75]. As ecosystems are complex and made of many inter-connected relationships, service provision often depends on habitat connectivity and environmental factors. For example, kelp primary productivity varies with light availability [97], while holdfast size and shape influence associated biodiversity [31, 32, 116]. Additionally, oxygen production will vary with light exposure (which can be greatly affected by water clarity and turbidity), and wave attenuation capacity (by kelp with the same abundance) declines with increasing depth [44, 54, 81, 110]. The adoption of ES in marine spatial management necessitates spatial estimates of service provision and an understanding of how these may be driven by biophysical conditions. Previous research has drawn complex biophysical relationships with service provision into manageable principles [132], facilitating framework development that can be utilized in the assessment and decision-making process. Similar approaches have been applied to urban environments (Forman 2016), deep-sea ecosystems [68], and shellfish habitats [105]. For example, [106] quantified four ES from estuarine bivalves in Tauranga Harbour, New Zealand. Utilizing knowledge of the provision of services, obtained by a systematic review, this research created spatial estimates of bivalve ES provision for conservation practise [105, 106]. This methodology is transferable to other species and locations, supporting spatial management needs [105, 106, 132].

To expand this approach to mapping kelp ES provision, accurate spatial mapping of kelp ES provision requires robust data on distributions of kelp abundance, which can be estimated using Species Distribution Models (SDMs) [18, 20]. While ES provision is strongly linked to kelp abundance [3, 39] and morphology [22, 140], it is also mediated by biophysical drivers such as light availability, water depth, and turbidity. However, the combined effects of abundance and biophysical drivers on ES remain poorly quantified [36, 63] and continuous spatial data over large scales relevant for management remain sparse or limited to estimates of occurrence of kelp rather than abundance or measurements of biomass and kelp productivity, limiting ES integration into management tools like marine spatial planning [83, 89, 132].

In the northeast Atlantic, seven habitat-forming kelp species are known to occur [112]. Among these, *Laminaria hyperborea* and *Saccharina latissima* are two of the most widespread. These species exhibit two growth forms, stipitate and prostrate, respectively, with distinct morphological adaptations that differentially influence ecological function and ES provision. Stipitate kelps have a rigid, upright stipe that elevates the blade in the water column optimizing light capture and nutrient uptake [50, 131]. In contrast, prostrate kelps grow horizontally along the substrate, forming canopies that reduce understory light but enhance sediment stabilization [40]. This dichotomy influences competitive outcomes, with stipitate forms often dominating in high-flow environments, while prostrate species may thrive in sheltered or disturbed habitats [29]. Exploring whether these growth strategies results in different associated ES, or ES provision remains an evidence gap [46].

*Laminaria hyperborea* (tangle kelp or curvie) is a perennial (living up to 18 years), stipitate kelp dominant on subtidal rocky reefs in the NE Atlantic, ranging from Svalbard in Norway to the Iberian Peninsula [12] at depths of 0.5–15 m, with the deepest records ~ 50 m in clear waters [21, 76]. With its large canopy and erect stipes, it forms forests which host high epiphytic diversity and provide habitat for commercially important species such as lobster and juvenile cod [4, 59, 91, 126]. Forests of *Laminaria hyperborea* have been shown to contribute substantially to carbon storage, with biomass estimates exceeding 200 tons wet weight per hectare [103]. Their slow decomposition rate also promotes detrital food webs, while their canopies dampen wave energy, reducing coastal erosion [112]. This kelp species is not found in areas influenced by sediment scour and is further absent in extremely exposed or sheltered areas [21].

*Saccharina latissima* (sugar kelp) is a fast-growing, prostrate kelp species widely distributed across temperate northern hemisphere coasts, from the northeast Atlantic to the northwest Pacific at depths from ~ 0.5–30 m [23, 25] with records at 30–35 m depth more common in clear Norwegian and Icelandic waters [51]. Bartsch et al., [14]. In extreme cases it has been found at ~ 50 m depth [74], with more efficient photosynthesis under low light conditions than *L. hyperborea* [19, 38]. It thrives in sheltered to moderately exposed environments, where it can form dense stands in subtidal zones enhancing biodiversity by providing habitat for epifauna and nursery grounds for fish [35]. This kelp is a key primary producer, contributing significantly to carbon storage between 0.7 and 1.2 kg C m⁻² yr⁻¹ [48, 99] and nutrient cycling in coastal ecosystems [41]. Additionally, *S. latissima* is commercially valuable for its high polysaccharide content, supporting aquaculture and bioremediation efforts [24, 100]. Its potential use in bioremediation is linked to its physiological affinity for nitrogen storage, and its use in bioremediation would require cultivation at a much greater scale than is currently practised [24, 100]. Given the contrasting morphologies of these species, it is expected that they differ in ES provision. The stipitate form of *Laminaria hyperborea* is likely to support greater structural habitat complexity and associated biodiversity [110, 118]. In contrast, it is expected that the prostrate *Saccharina latissima* may contribute more to sediment stabilization, nutrient cycling and food production whereas it is often used in commercial kelp growing [145, 146].

While prior reviews have assessed kelp ES globally [43, 111] and locally [36, 112], or valued individual services [82, 112], to date, no systematic review or map has specifically examined ecosystem service provision in the two focal kelp species, *Laminaria hyperborea* and *Saccharina latissima*, despite their ecological importance and distinct growth forms. In addition, to advancing approaches in estimating kelp ES, no study has systematically linking abundance and/or biophysical drivers to ES provision. For example, although holdfast biodiversity depends on both substrate composition and wave dynamics (Christie et al., 2003; [125, 126]), the relationships remain unintegrated into a principle or framework.

### Stakeholder involvement/expert elicitation

This systematic map is being led by marine ecologists with expertise in kelp mapping and ecology, ecosystem services, marine science, biodiversity, ecological policy and conservation management. After explaining the overall research question to the wider team and outlining the exclusion criteria (Table 2), a focus on the growth form of kelp, stipitate or prostrate was suggested and included. Following this input, research questions were collated, and initial search terms proposed based on the literature and existing knowledge. These search terms were then circulated to experts from the team’s network, with expertise in kelp forest ecology, marine climate change impacts, and ecosystem service valuation that informed the final search terms.

### Objective of the review

This systematic map’s primary aim is to document and classify the evidence describing ecosystem service provision by *Laminaria hyperborea* and *Saccharina latissima*, and to identify gaps and biases in the evidence base between the two species (which represent contrasting growth forms). A secondary aim is to map the evidence linking ecosystem service provision to variation in biophysical drivers (e.g. temperature, turbidity, depth, wave exposure) including the study designs and types of evidence used.

Accordingly, the primary question of this systematic map is: What ecosystem services have been reported for *Laminaria hyperborea* and *Saccharina latissima?* The secondary questions are: What evidence exists on associations between biophysical drivers and ecosystem service provision for these species? What types of evidence (quantitative and/or qualitative), and what study designs (e.g. spatial contrasts, temporal contrasts, experiments, gradient analyses), have been used to assess ecosystem service provision and driver relationships?

The primary mapping question is structured using a PO framework (Population, Outcome). In this instance, search terms and eligibility criteria were linked to Population = Kelp species: *Laminaria hyperborea* and *Saccharina latissima*, and Outcome = any direct measurement of ecosystem service provision or a clearly defined proxy linked to an ecosystem service. Within eligible articles, a coding approach will be used to answer our secondary questions.

## Methods

This protocol follows the reporting standards for systematic maps syntheses (ROSES) for review protocols (Additional file 1) [62].

### Searching for articles

The systematic map will search for literature through two main bibliographic sources, Scopus and Web of Science Core Collection, as well as one online search platform, Google Scholar. Full search strings, date/time of execution (UTC), and hit counts will be provided in supplementary material. We expect to find most studies from the Web of Science Core Collection and Scopus based on the initial scoping of the topic area. Furthermore, we created a benchmark list containing ten articles that provide evidence on the biophysical drivers of ES provision of kelp, which was used to test the comprehensiveness of the proposed search string. Following execution in the specified bibliographic sources, the final search string successfully identified all benchmark articles, confirming its appropriateness for addressing the review question. Web-based searches will also be conducted on Google Scholar as a supplementary source using English search terms. Full-text articles were accessed using the institutional subscriptions of Newcastle University library.

### Search strings

Search strings were created based on Boolean methods using the key terms identified, such that an article will be returned if it referred to kelp (and its synonyms), a type of ecosystem service and/or a biophysical driver. These combinations used search rules connecting terms using “AND”, and synonyms within subject terms connected using “OR”. We searched using a combination of different synonyms and tested them against a list of ten benchmark articles. In this study, the minimum number of terms that give both high sensitivity and low specificity will be used as the final search string (Additional file 2). The final, comprehensive search string utilized in this protocol (presented below and in Additional file 2) was formatted using the Web of Science Core Collection syntax. This master string was then carefully adapted for all other bibliographic sources (including Scopus and Environmental Sciences and Pollution Management) to ensure accurate and appropriate indexing across platforms. The search strategy and search strings were discussed and improved upon with the help of an information specialist (Newcastle University Library).

The final search string was then adjusted accordingly to the syntax of other bibliographic sources. Due to resource constraints, this systematic map will be restricted to publications in English, including literature with abstracts published in English. Searches within the Web of Science Core Collection were specifically restricted to the following indexes to ensure comprehensive coverage: Science Citation Index Expanded (SCI-EXPANDED), Social Sciences Citation Index (SSCI), Arts & Humanities Citation Index (A&HCI), Emerging Sources Citation Index (ESCI), Conference Proceedings Citation Index – Science (CPCI-S) and Conference Proceedings Citation Index – Social Science & Humanities (CPCI-SSH). The final search string was refined within the Web of Science Core Collection by testing it against ten benchmark papers (Additional file 3). Additionally, Web of Science Core Collection will be searched using the Topic (TS) field, which captures the title, abstract, and keywords. Below are the final search strings formatted as for Web of Science:

(TS=(“Laminaria hyperborea” OR “tangle kelp” OR “Saccharina latissima” OR “Laminaria saccharina” OR “sugar kelp” OR stipitate OR prostrate OR “kelp forest*”) )

AND.

(TS=(“biomass production” OR “algin* extract*” OR biofuel* OR aquacult* OR “food source*” OR fertili* OR “carbon sequest*” OR “carbon stor*” OR “blue carbon” OR “nutrient cycl*” OR “nitrogen remov*” OR “oxygen produc*” OR “wave attenuat*” OR “erosion control” OR “coastal protect*” OR “storm buff*” OR “water qual* improv*” OR “pollut* remov*” OR “heavy metal uptake” OR biodivers* OR “species rich*” OR “habitat provis*” OR “nursery ground*” OR refug* OR “trophic support” OR “food web*” OR “primary product*” OR ecotourism OR recreation* OR diving OR education* OR “aesthetic value” OR “cultural signific*” OR “ecosystem service*” OR “natural capital” OR “people* connection*” OR nutrient* OR growth* OR light*) )

### Web-based search

Our web-based search using Google Scholar will help identify- potentially relevant studies that may have been missed by database searches, as well as relevant grey literature. Grey literature will include government agency reports (e.g. Environment Agency, JNCC, or regional marine authorities), academic dissertations and thesis, and reports from major environmental NGOs. All retrieved documents will be assessed for eligibility according to the same PO based inclusion and exclusion criteria. The final search terms for all platforms are provided in Additional file 2.

### Comprehensiveness and validation of the search strategy

To ensure the comprehensive and sensitivity of the final search string, a validation exercise was performed. A list of 10 highly relevant benchmark articles that directly address the review question (Smith et al. 2018, Jones 2020) was compiled prior to the final search execution (Additional file 3). It was the successful retrieval of these known benchmark articles that was used as the primary metric to estimate the comprehensiveness of the search strategy. Following execution in the bibliographic sources, the final search string successfully identified all benchmark articles (Additional file 3). This confirmed the appropriateness of the search terms, field codes, and Boolean logic for capturing the key literature on kelp species and ecosystem services. These search terms were then further adapted for the other search engines used (Additional file 2).

### Article screening and study eligibility criteria

Due to the resources available and the dominance of English-language publication in marine ecology, this systematic map will be limited to English sources. This may introduce some geographic bias, particularly underrepresenting studies from South America. Based on the elements of the questions, we identified key terms that refer to PO (Population, Outcome) framework to design the criteria [65] (Table 2). Structured screening will be undertaken to ensure that only relevant studies are included in the systematic map. This screening process will be conducted by the review team using pre-defined criteria for inclusion/exclusion, following the Collaboration for Environmental Evidence (CEE) guidelines [1]. We will create the ROSES flow diagram by using the ROSES flowchart R package to report the number of papers excluded at each screening stage [61]. In this instance, Population = Kelp species: *Laminaria hyperborea* and *Saccharina latissima and* Outcome = any direct measurement of ecosystem service provision or a clearly defined proxy linked to an ecosystem service.

#### Screening process

To ensure a transparent and replicable screening process as best possible, all identified articles will be managed in the different screening stages:

1. Remove duplicates: We will use R package ‘Revtools’ [142] to remove duplicate items from search results based on the match between the title and DOI. Subsequently, we will further remove duplicates using duplicate resolution feature on the Rayyan website [95].
2. Title and abstract screening: In this step, two independent primary reviewers will screen the titles and abstracts of all retrieved studies against the eligibility criteria (Table 2). Any paper that does not meet the eligibility criteria will be excluded. Publications for which relevance cannot be determined by the screening of titles and abstracts will be retained for the next screening step. In situations where there are discrepancies between reviewers during the title and abstract screening phase, or for articles that are coded ‘maybe’, a first attempt will be made to resolve this through discussion between the two reviewers. If this fails to resolve the discrepancy, a third reviewer will make the final decision.
3. Full text screening: publications will be subject to a full-text reading and assessed according to the eligibility criteria of the systematic map (Table 2). This screening step will be recorded in an Excel spreadsheet where publications are provided with a study ID number, and information including title, author, publication year, and journal. Publications will be marked ‘Include’, ‘Exclude’ and ‘Maybe’ against Study type, Population (P) and Outcome (O). Exposure (E) and Comparator (C) will be coded where present to support the driver-focused subset and will not be used as exclusion criteria for the primary map. The publications that meet all eligibility criteria coded: “Include” will be included in the analysis and synthesis. Publications that fail to meet one or more of the eligibility criteria coded: “Exclude” will not be included in the analysis. The same as step 2, any disagreement between the two reviewers or articles that are coded “Maybe” will be resolved through discussion between the reviewers. If no agreement can be reached, a third reviewer will make the final decision.

To further the reliability and repeatability of the eligibility criteria, Cohen’s Kappa test will be used. Cohen’s Kappa test is a statistical measurement used to assess the level of agreement between two reviewers that accounts for the possibility of agreement occurring by chance [33]. It will show the consistency of the screening results for the two primary reviewers. A value of over 0.6 indicates the screening results and eligibility criteria are reliable and reproducible.

Cohen’s Kappa will be used for a mandatory calibration exercise prior to the full-scale screening process. The two primary reviewers will independently screen a random, non-overlapping subset of 10% of the retrieved titles and abstracts. The level of agreement will be calculated using Cohen’s kappa. A minimum threshold for substantial agreement is set at 0.6. If the calculated score is below 0.6, the reviewers will halt the main screening process. They will then meet to discuss and reconcile all disagreements, clarify the interpretation of the eligibility criteria, and refine the screening rules. The threshold of 0.6 was selected as it represents the entry point for ‘substantial agreement’ according to the widely accepted Landis and Koch (1977) scale. This ensuring that the inclusion/exclusion criteria are being applied consistently and objectively across multiple reviewers before the full screening process commences. This calibration cycle (screening a new subset, calculating kappa score and resolving differences) will be repeated until the minimum threshold is achieved. Once this substantial agreement is demonstrated, the full-scale, independent screening will commence. Authors will not screen or critically appraise any studies they have been directly involved in. Furthermore, to ensure procedural independence and avoid selection bias, a conflict-of-interest procedure will be strictly followed. Any reviewer who is an author on a retrieved article will be excluded from the eligibility screening and data extraction process for that specific article. A third, independent reviewer will then screen and extract data for that conflicted article. If no reviewers have authored articles included in the final review, a statement of this fact will be included in the final report.

#### Eligibility criteria

Ensuring consistency and transparency in the study selection, this systematic map will use the PO (Population, Outcome) framework to design the criteria [65], as presented in Table 2. A detailed list of all the articles included and excluded at the full-text stage screening process will be provided. This list will include the reason for the articles’ exclusion in this review. Furthermore, a flow diagram (following ROSES guidelines) will be used to record the number of articles identified, screened, and included in the review. For articles that progress to the full-text screening stage but are subsequently excluded, a comprehensive list of these articles will be provided in the final review, clearly stating the primary reason for exclusion for each study.

**Table 2:** Inclusion and exclusion criteria using a PO framework to answer the primary mapping question

Component	Inclusion criteria	Exclusion criteria
**Study type** The type of scientific study design and literature type included.	- Primary empirical studies (field based, laboratory based, and modelling studies). - Grey literature, including government reports, technical reports, and postgraduate thesis/dissertations. - Secondary syntheses, including narrative reviews, literature reviews, and systematic reviews. - Peer reviewed and non-peer reviewed sources.	- Book chapters, editorials and opinion pieces. - Conference abstracts without full papers. - Purely modelled studies with no primary data involvement or collection.
**Population (P)** The species or ecosystem being studied.	- Studies that focus on the two target kelp species (a *Laminaria hyperborea* and *Saccharina latissima* ). - Study sites can be located within temperate or polar areas with a focus on the two species of interest, *Laminaria hyperborea* and *Saccharina latissima* **.**	- Studies which focus purely on a different species that are not,* Laminaria hyperborea* or *Saccharina latissima* **.** For instance, a study just of *Laminaria digitata* ** .**
**Study outcome (O)**	Studies that provide a direct measurement or a clearly defined inference of ecosystem services (including provisioning, regulating, and cultural services). This can include measurements of kelp forest characteristics (e.g. abundance, biomass, or structure) but only if they can be linked to the delivery of an ecosystem service.	Studies that purely measure kelp characteristics (e.g. primary productivity, abundance, density, or stipe length) that cannot be used in inference to ecosystem service provision. Studies focused solely on cultural services without a defined measurement or descriptive outcome are also excluded.

### Data extraction and coding strategy

Data extraction and coding will be undertaken in the designed review data sheet (Additional file 4). The data sheet was developed in a pilot test using the benchmarked articles as well as additional conference between the two literature reviewers and the review moderator. This data extraction sheet allows for a uniform coding for data extraction in the review. Reviewers will extract and code data to map the studies’ general information after removing duplicates (step one) has been undertaken on a subset of identified relevant papers that remain. The recorded information will include the species focused on, the geographical area, study design, and the results of the study (Additional file 4). The research details are mainly focused on the study area and the ecosystem services identified in the study which can be linked to service provision. The primary purpose of this systematic map is to document and synthesise the evidence describing ecosystem service provision by two morphologically distinct kelp species (*Laminaria hyperborea* and *Saccharina latissima*). Secondary analyses will map how ecosystem service provision has been examined in relation to biophysical drivers for each species, including the types of evidence, methods, and study designs used.

A PECO type approach (Population, Exposure, Comparator, Outcome) will be used to structure coding for the biophysical driver-focused subset of studies (Additional file 4). That is, from within the studies included using the PO framework, we will extract data regarding biophysical drivers (Exposure) and study designs (Comparator). The review map will classify Exposure by the presence of any biophysical drivers e.g. (turbidity, temperature, light availability, depth). This includes measurements from satellite data, laboratory experiments, or in-situ recordings. It will further classify Comparator by recording the type of comparison used (e.g., spatial, temporal, experimental, or gradient-based) and the direction of comparison. This includes contrasts such as exposed vs. sheltered sites, before vs. after storm events, or laboratory control groups.

To ensure the accuracy and consistency of the data extraction process, two reviewers will independently extract data from the ten benchmark articles. These articles, which were initially used to validate search comprehensiveness, now serve as a training set for the extraction phase. Any disagreements in the data extracted from these ten papers will be discussed and resolved to refine the coding definitions before the full extraction begins. Data on the ES provided by kelp including general data on experiment location, year, whether it is quantitative or inferred, will be extracted from these ten selected papers by two reviewers and any disagreements will be reviewed and resolved through discussion. If disagreements persist, they will be resolved by a third reviewer. When information is present in tables or graphs, all information will be extracted; if it is not possible to interpret the information from graphs, the corresponding author will be contacted via email, and if no response is received within three weeks, the data point will be marked as ‘not reported’ in the database. Data extraction will prioritise ecosystem service type, how service provision is measured or inferred, and which of the two focal species is studied. Where reported, we will also code biophysical drivers and the type of comparison / analysis used to relate drivers to service provision, enabling a structured map of the biophysical driver-focused subset. All extracted data will be available as supplementary information in the systematic map.

### Study mapping and presentation

Data will be synthesised narratively and through descriptive statistics to provide a comprehensive overview of the evidence base. In accordance with systematic mapping methodology, no quantitative meta-analysis will be performed, with the objective to map the distribution and extent of research.

Findings will be presented using a ROSES flow diagram to detail the study selection process. The synthesis will include descriptive mapping showing trends in research volume mapped temporally (by year of publication) and geographically (by country or region of study). This will identify the historical trajectory of kelp ecosystem service (ES) research and highlight regions where evidence is concentrated or lacking. Additionally, bar charts will visualise the frequency of studies linking the two target species (*Laminaria hyperborea* and *Saccharina latissima*) to specific MEA service categories (Provisioning, Regulating, Supporting, and Cultural). This approach is designed to identify areas of high knowledge and contrast them against under-represented services. Finally, we aim to discuss knowledge gaps within the evidence base. This includes identifying imbalances between the two target species and assessing the breadth of research across all four MEA categories, with a particular focus on the potential scarcity of research across species and service type. Similarly to our focus on MEA categories, our secondary question focuses on the biophysical drivers affecting these services, which we aim to visualise with heatmaps that illustrate the intersections between drivers and ecosystem service outcomes.

## Supplementary Information

Below is the link to the electronic supplementary material.

Additional file 1

Additional file 2

Additional file 3

Additional file 4

## Data Availability

All data generated or analysed from the pilot testing are included in this published article and its Additional files.
