# Supplementary material for: Characterising ecosystem service provision by two morphologically distinct kelp species (Laminaria hyperborea and Saccharina latissima) and the biophysical drivers shaping these services: a systematic map protocol
Source: Environ Evid. 2026 Apr 21;15:6. doi: 10.1186/s13750-026-00385-w (PMC13255359; doi:10.1186/s13750-026-00385-w)
Supplement: Supplementary file 3 — Additional file 3 [file 13750_2026_385_MOESM3_ESM.pdf]

Item number	Section / sub-section	Topic	Description	Further explanation	Checklist/Meta-data	Author response	Comments
1	Title	Title	review update protocol...".	question.	Meta-data		
2	Type of review	Type of review	systematic review from a systematic map	See CEE Guidance on amendments and updates [1]	Meta-data	systematic review from a systematic map	
3	Authors contacts	Authors contacts	The full names, institutional addresses, and email addresses for all authors must be provided.		Checklist	Yes	
4	Abstract	Structured summary	including the review question; 2) Methods, how the review will be conducted and the outputs that are expected (specifically		Checklist	Yes	
5	Background	Background	necessary and what it aims to contribute to the field.	the intervention or exposure to the outcome.	Checklist	Yes	
6	Stakeholder engagement	Stakeholder engagement	The planned/actual role of stakeholders throughout the review process (e.g. in the formulation of the question) must be described and explained (using a broad definition of 'stakeholder', including e.g. researchers, funders and other decision-makers; see [2])		Checklist	No	
7	Objective of the review	Objective	Describe the primary question and secondary questions (when applicable).	questions are usually linked to sources of heterogeneity (effect modifiers).	Checklist	Yes	
8		components	Break down and summarise question key elements e.g. population, intervention(s)/exposure(s), comparator(s), and outcome(s).	For other question types see [3,4]	Meta-data	PECO	
	Methods						
9	Searches	Search strategy	subscribed for each database), search options (e.g. 'topic words' or 'full text' search facility), efforts to source grey literature,	Details regarding search strategy testing should be provided.	Checklist	Yes	
10		Search string	Provide Boolean-style full search string and state the platform for which the string is formatted (e.g. Web of Science format)		Meta-data	Provided	
11		databases	List languages to be used in bibliographic database searches.		Meta-data	Yes - English only	
12		Languages – grey literature	List languages to be used in organizational websites searches and web-based search engines.		Meta-data	Yes - English only	
13		Bibliographic databases	Provide the number of bibliographic databases to be searched.		Meta-data	Yes - 3 (No grey lit yet)	
14		Web – based search engines	Provide the number of web – based search engines to be searched.		Meta-data	Yes - 1 (Google Scholar)	
15		Organisational websites	Provide the number of organisational websites to be searched.		Meta-data	None - currently	
16		of the search	Describe the process by which the comprehensiveness of the search strategy was assessed (i.e. list of benchmark articles).		Checklist	Yes	Yes 10 benchmark articles
17		Search update	Describe any plans to update the searches during the conduct of the review.	performed more than two years prior to review completion.	Checklist	Yes	
18	inclusion criteria	Screening strategy	Describe the methodology for screening articles/studies for relevance/eligibility.		Checklist	Yes	
19		Consistency checking	undertaken and estimated proportion of articles/studies that will be screened and checked for consistency by two or more		Checklist	Yes	
20		Inclusion criteria	question key elements (e.g. relevant subject(s), intervention(s)/exposure(s), comparator(s), outcomes, study design(s)) and any		Checklist	Yes	
21		Reasons for exclusion	State that you will provide a list of articles excluded at full text with reasons for exclusion.		Checklist	Yes	
22	Critical appraisal	Critical appraisal	evidence base as a whole).		Checklist	Yes	
23		Critical appraisal strategy	Describe how the information from critical appraisal will be used in synthesis.		Checklist	Yes	
24		Consistency checking	Describe how repeatability of critical appraisal of study validity will be tested.		Checklist	Yes	
25	Data extraction	strategy	if variables to be extracted as meta-data and those that will be coded).		Checklist	Yes	
26		Data extraction strategy	(ideally piloted))		Checklist	Yes	
27		Approaches to missing data	Describe any processes for obtaining and confirming missing or unclear information or data from authors.		Checklist	Yes	
28		Consistency checking	Describe how repeatability of the meta-data/data extraction process will be tested.		Checklist	Yes	
29	for heterogeneity	for heterogeneity	provide details of how the list was compiled (including consultation of external experts).	thought to be most important and amenable to analysis.	Checklist	Yes	
30	Data synthesis and presentation	Data synthesis and presentation	qualitative, narrative, qualitative and quantitative, narrative and mixed-methods)		Meta-data	Narrative and qualitative	
31		Narrative synthesis strategy	any map databases) and figures.	their findings) must be avoided. Must include a summary of the outputs of	Checklist	Yes	
32		Quantitative synthesis strategy	[illegible] complex data, statistical methods for combining data from individual studies, and any planned exploration of heterogeneity (e.g. sensitivity analysis, subgroup analysis and meta-regression). If all studies may not be selected for synthesis explain criteria for selection (e.g. incomplete or missing information).	Compulsory if appropriate for data	Checklist	n/a	Data may not be appropriate for a quantative assessment (see section on potential outcomes)
33		Qualitative synthesis strategy	plan to analyse subgroups/subsets of data. If all studies may not be selected for synthesis explain criteria for selection (e.g.	Compulsory if appropriate for data	Checklist	Yes	
34		Other synthesis strategies	methods) and justify your methodological choice.	Compulsory if appropriate for data	Checklist	Yes	
35		Assessment of risk of publication bias	Describe planned methods for examining the possible influence of publication bias on the synthesis.	For quantitative syntheses this may be done using diagnostic plots or statistical tests	Checklist	systematic review from a systematic map	
36		strategy	that warrant further primary research).	Optional	Checklist	No	
37		independence	regarding inclusion or critical appraisal of their own work.	should be prevented from unduly influencing inclusion decisions, for	Checklist	Yes	
38	Declarations	Competing interests	Describe of any financial or non-financial competing interests that the review authors may have.		Checklist	Yes	

References

[1] Bayliss, H.R., Haddaway, N.R., Eales, J., Frampton, G.K. and James, K.L., 2016. Updating and amending systematic reviews and systematic maps in environmental management. Environmental Evidence, 5(1), p.20.

[2] Haddaway, N.R., Kohl, C., da Silva, N.R., Schiemann, J., Spök, A., Stewart, R., Sweet, J.B. and Wilhelm, R., 2017. A framework for stakeholder engagement during systematic reviews and maps in environmental management. *Environmental Evidence* , *6* (1), p.11.

[3] Collaboration for Environmental Evidence. 2018. Guidelines and Standards for Evidence synthesis in Environmental Management. Version 5.0. www.environmentalevidence.org/information-for-authors.

[4] Leeds Institute of Health Sciences. https://medhealth.leeds.ac.uk/info/639/information_specialists/1500/search_concept_tools. Accessed 12/11/2017.
